# Left atrial appendage closure in conjunction with radiofrequency ablation: Effects on left atrial functioning in patients with paroxysmal atrial fibrillation

**DOI:** 10.1515/med-2024-0951

**Published:** 2024-04-12

**Authors:** Jing Lv, Rui Wang, Jing Yang, Ling You, Chao Yang, Yan Zhang, Qian Liu, Lei Yin, Jin-ting Liu, Rui-qin Xie

**Affiliations:** Division of Cardiology, The Second Hospital of Hebei Medical University, Xinhua District, Shijiazhuang, Hebei, 050051, China; Division of Cardiology, Xingtai People’s Hospital of Hebei Medical University, Xingtai, Hebei, 054000, China; Division of Cardiology, The Second Hospital of Hebei Medical University, 215 Heping West Road, Xinhua District, Shijiazhuang, Hebei, 050051, China

**Keywords:** catheter ablation, left atrial appendage closure, left atrial function, paroxysmal atrial fibrillation, speckle tracking echocardiography

## Abstract

**Objective:**

In the present study, we investigated the impact of left atrial appendage closure (LAAC) following catheter ablation (CA) on the left atrial structure and functioning of patients with paroxysmal atrial fibrillation (AF).

**Methods:**

Patients with paroxysmal AF were enrolled in this single-center prospective cohort study between April 2015 and July 2021; 353 patients received CA alone, while 93 patients received CA in combination with Watchman LAAC. We used age, gender, CHA2DS2-VASc, and HAS-BLED scores as well as other demographic variables to perform propensity score matching. Patients with paroxysmal AF were randomly assigned to the CA combined with Watchman LAAC group (combined treatment group) and the simple CA group, with 89 patients in each group. The left atrial structure, reserve, ventricular diastole, and pump functions and their changes in patients were assessed using routine Doppler echocardiography and 2D speckle tracking echocardiography over the course of a 1-year follow-up.

**Results:**

At 1-week follow-up, the reserve, ventricular diastole, and pump functions of the left atrium (LA) increased in both groups; these functions were gradually restored at the 1- to 3-month follow-up; they were close to or returned to their pre-operative levels at the 3-month follow-up; and no significant differences were found compared with the pre-operative levels at the 12-month follow-up. In the first 3 months, the reserve (Ƹ, SRs) and pump functions (SRa) in the combined treatment group decreased significantly when compared with the simple CA group, and the differences were statistically significant.

**Conclusion:**

Patients with paroxysmal AF may experience a short term, partial effect of LAAC on LA reserve and pump functions, which are gradually restored and the effect disappears by 12 months.

## 1 Introduction

As a leading cause of both stroke and heart failure, atrial fibrillation (AF) is a serious issue affecting the modern world [1,2]. Patients with AF have a number of alternatives for stroke prevention; one of the options is left atrial appendage closure (LAAC), which has been shown to be as effective as, if not more successful than Warfarin and novel oral anticoagulants in avoiding ischemic stroke [3,4]. Thus, AF ablation plus LAAC (termed “one-stop surgery”) can safely and effectively restore sinus rhythm in the treatment of heart failure and prevention of stroke [5]. Under normal conditions, the left atrial appendage (LAA) performs both mechanical and endocrine roles; however, AF causes a dramatic loss in the mechanical performance of LAA, with the rate of decline differing between paroxysmal and persistent AF [6–8]. Morphology of the LAA can significantly affect pathophysiological properties of the LAA, choice of closure technique, the course of the procedure, its results, and complications. Presented morphometrical data should be considered during planning and when performing procedures targeted to LAAC, as significant differences in the anatomy of the LAA may be encountered in patients with AF. The study of Słodowska et al. confirmed that substantial morphometrical differences in LAA orifice are found, which is significantly larger and more oval in patients with AF compared to healthy controls. Although no difference in LAA body type and size is observed, the LAA ejection fraction is significantly lower in AF patients [9]. Consequently, the effect of LAAC on the left atrial structure and functions of AF patients, especially those with different types of AF is still unclear.

According to our previous studies, research on the left atrial functions of patients with persistent AF confirmed that additional LAAC had an impact on the reserve and ventricular diastole functions of left atrium (LA) within 3 months [10], with no effect on the improvement of the LA functions after catheter ablation (CA) within a year. However, no research has systematically discussed the impact of the one-stop surgery on the LA functions of patients with paroxysmal AF. In light of the substantial disparities in the LAA functions between paroxysmal AF and persistent AF, it is hypothesized that LAAC has different effects on the LA functions of patients with paroxysmal AF and with persistent AF. LAAC provides excellent results in patients with AF. LAAC is recommended for the treatment of patients with AF in whom oral anticoagulation is contraindicated and the number of procedures is increasing every year [11]. Moreover, the indications for LAAC may be expanded in the future based on the encouraging results of some clinical trials [12,13]. This study uses patients with AF treated with radiofrequency ablation alone as a control group to explore whether additional LAAC on top of CA has an impact on the left atrial function in patients with paroxysmal AF. This may provide some references for clinicians to understand the concept of a one-stop surgery, and also offer more insights into selecting the best treatment strategy for LAAC therapy.

## 2 Methods

### 2.1 Study population

**Figure 1:**
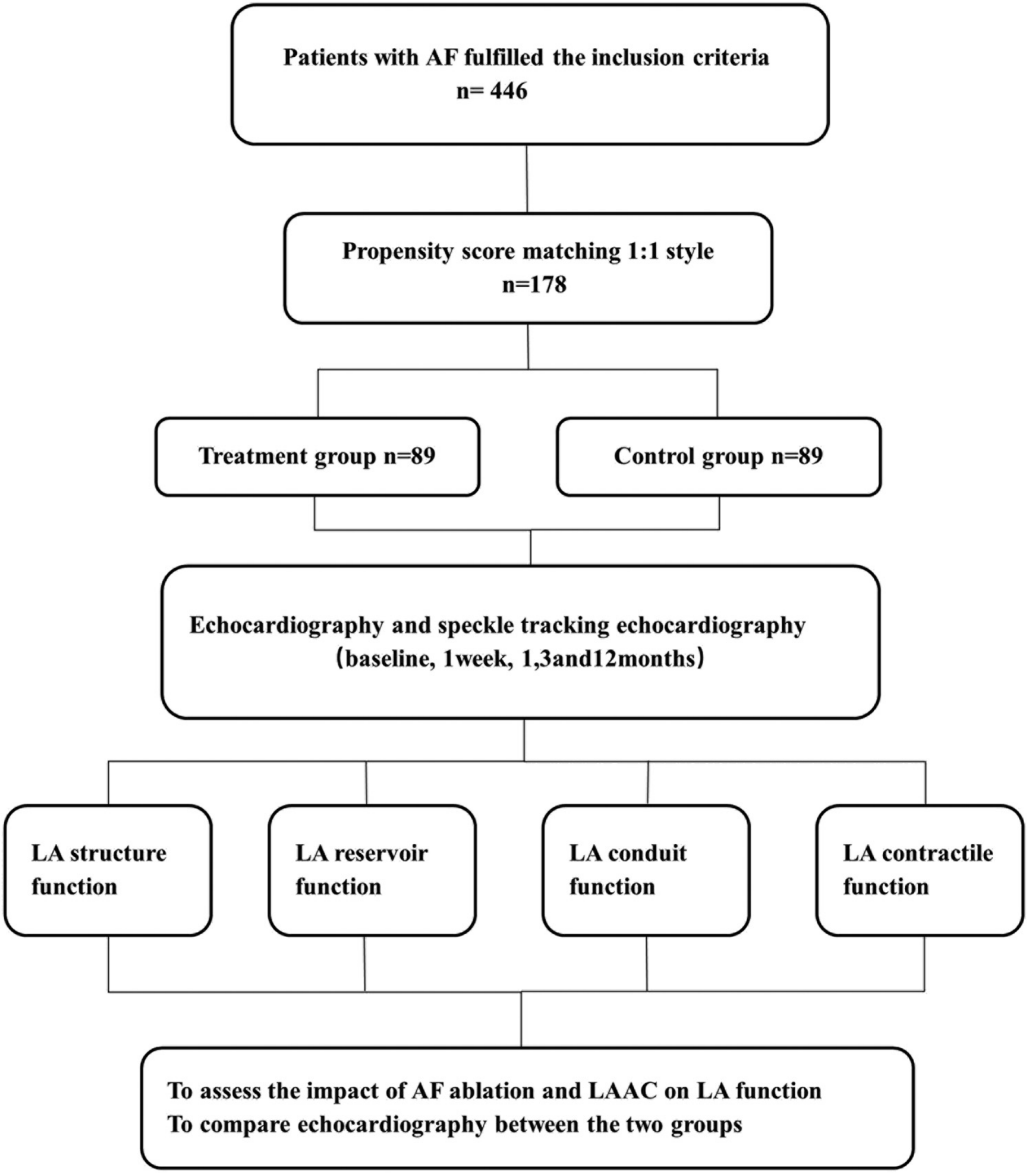
Flowchart of the study procedure. LA, left atrial; LAAC, left atrial appendage closure; AF, atrial fibrillation; treatment group; combined treatment group; control group; simple CA group.


In this single-center prospective cohort study, patients with paroxysmal non-valvular AF (NVAF) were selected and hospitalized in the Cardiology Department I of the Second Hospital of Hebe Medical University from April 2015 to July 2021. Paroxysmal AF is defined as AF that terminates spontaneously or with intervention within 7 days of onset. In this study, 93 patients with paroxysmal AF who were treated by CA combined with Watchman LAAC were included in the combined treatment group. This study exclusively employs endocardial LAAC for all cases. Inclusion criteria were (1) patients aged >18 years, (2) patients with symptomatic NVAF intractable with antiarrhythmic drugs, (3) patients with a high stroke risk (CHA2DS2-VASc score ≥2 points for male and ≥3 points for females), (4) patients with the HAS-BLED score ≥3 points or major hemorrhage events during anticoagulation treatment (severe organ hemorrhage such as cerebral hemorrhage), with a higher hemorrhage risk predicted, and (5) LAAC was preferred instead of long-term oral anticoagulants. Exclusion criteria included: (1) patients with LA or LAA thrombosis, (2) patients with LA enlargement (≥55 mm), (3) patients with severe valvular heart disease, (4) patients with serious sequelae after severe disabling ischemic stroke on the basis of NVAF, and (5) patients with life expectancy <1 year. In the corresponding period, 353 additional patients with paroxysmal AF who only received CA were included in the control group. Inclusion criteria were patients aged ≥18 years, with symptomatic NVAF intractable with antiarrhythmic drugs. Exclusion criteria were the same as those for the combined treatment group. Propensity score matching (PSM) extension was used at a 1:1 ratio in terms of the age, gender, CHA2DS2-VASc score, and HAS-BLED score, and 89 patients were included in the combined treatment group and the simple CA group each. The research flowchart is shown in Figure 1.

**Ethical approval:** This study was conducted in accordance with the declaration of Helsinki. This study was conducted with approval from the Ethics Committee of The Second Hospital of Hebei Medical University (No. 2022-R134).
**Informed consent:** A written informed consent was obtained from all participants.

### 2.2 Procedure

One to two days before the procedure, a transesophageal echocardiography (TEE) is performed to exclude the presence of thrombi in the LA and LAA, and to evaluate the type (chicken wing type, cauliflower type, and arrowhead type), size, and depth of the LAA, as well as to measure the emptying velocity of the LAA. A pulmonary vein enhancement CT scan is conducted to assess the anterior–posterior diameter, left–right diameter, superior–inferior diameter of the LA, and the cross-sectional short axis of both pulmonary veins.

### 2.3 CA procedure

All procedures were performed while patients were under conscious sedation and local anesthesia. CA was performed in all cases with wide antral circumferential until the entrance and exit block was demonstrated for each pulmonary vein. With a three-dimensional electroanatomical mapping system (CARTO R^@^ 3; Biosense Webster, Irvine, CA, USA), the ablation catheter (Thermocool SMARTTOUCH; Biosense Webster) was inserted into the LA to perform radiofrequency ablation. The mapping catheter was used to record pulmonary vein potentials (Lasso^®^ NAV Eco; Biosense Webster). Circumferential ablation in both the left and right pulmonary veins was confirmed. No additional ablation lines or complex fractionated atrial electrogram ablation was performed in this study. Sinus rhythm was restored by either ablation or electric cardioversion.

### 2.4 LAAC procedure

**Figure 2:**
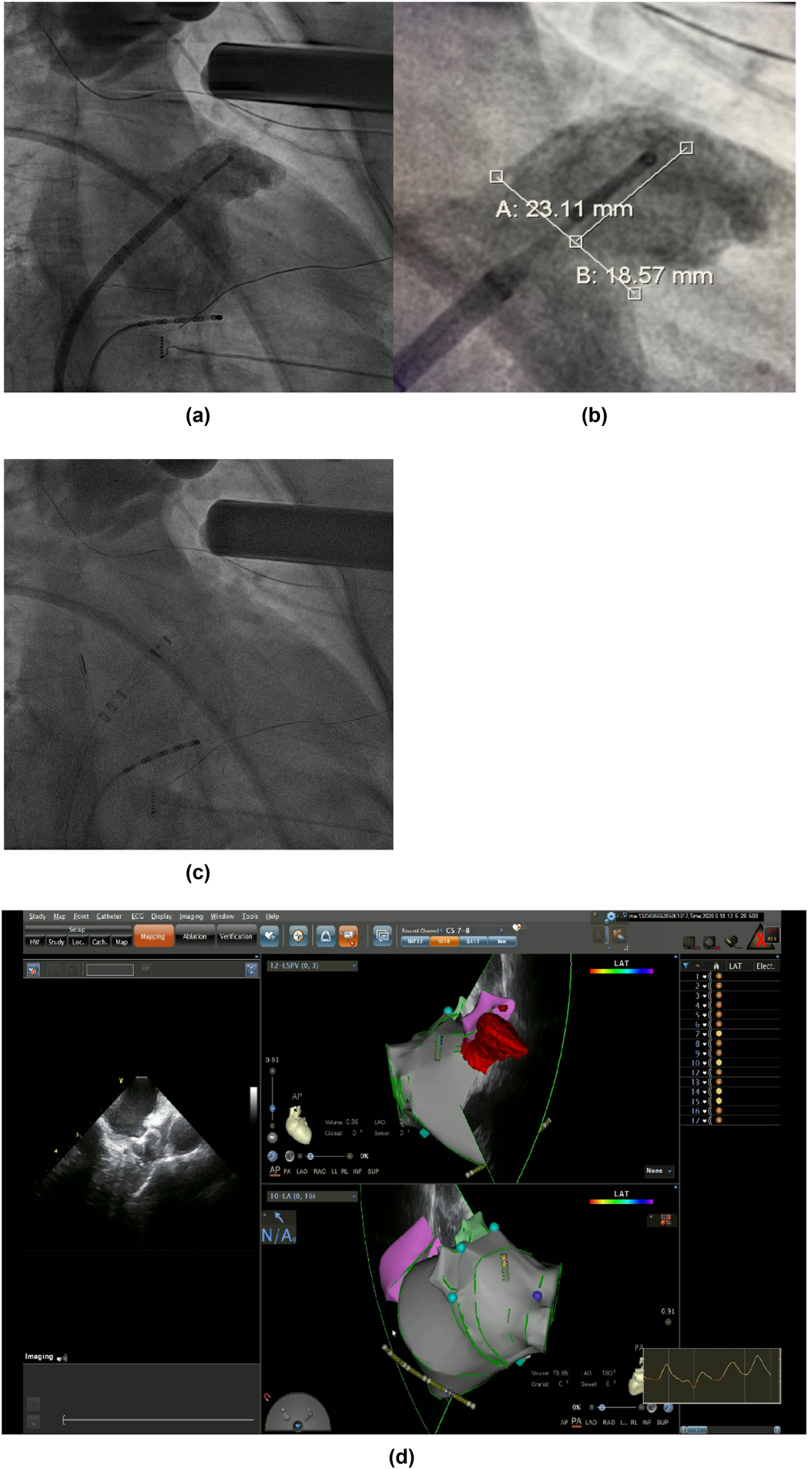
LAAC with WATCHMAN device. (a) and (b) Angiography of the LAA was performed to measure the ostial width and depth. (c) Fluoroscopic view after delivery of the WATCHMAN device in the proper position. (d) Peri-device leakage and compression were detected by TEE post-implantation.

Occluder devices for the LAA were implanted under fluoroscopic or TEE guidance in the combined treatment group as soon as pulmonary vein isolation (PVI) was completed. After CA, the LAA was occluded with the use of occluder devices (Watchman; Boston Scientific, Marlborough, MA, USA). All patients undergoing LAAC procedures received up to 1,500 mL of 0.9% normal saline intravenous infusion to ensure the filling of the LAA. The process of Watchman implantation is shown in Figure 2. Angiography of the LAA was performed to measure the ostial width and depth. Oversizing by 10–20% of the diameter of the LAA was recommended in order to choose an appropriate device size, and the device was released in the proper position after stability testing. Before releasing the occluder device, we ensured that no or minimal (<5 mm) residual flow remained and that adjacent structures (such as the mitral annulus and circumflex coronary artery) were not compressed.

### 2.5 Echocardiographic examination

The left atrial functions were determined using echocardiography (iE33 system, equipped with the X3-1 probe; Philips Medical Systems, Eindhoven, Netherlands) at baseline, 1 week, and 1, 3, and 12 months after surgery.

All patients at follow-up had sinus rhythm when 2D transthoracic echocardiography (2D-TTE) and speckle tracking echocardiography (2D-STE) was conducted. The LA volumes at three time points during the cardiac cycle were measured on the 2D-TTE: (1) at the diastasis before the bicuspid valve opened (maximum LA volume [LAVmax]), (2) the end-systolic frame before the bicuspid valve closed (minimum LA volume [LAVmin]), (3) the time of the P-wave on the echocardiography, or the last frame before the bicuspid valve opened (before atrial systole [LAVpreA]), and (4) the left atrial diameter (LAD) at the end of systole was measured on the parasternal long-axis view.

An experienced senior sonographer completed the measurement using speckle tracking technique on the Philips Qlab CMQ software based on 2D echocardiography. Three reference points were placed on the 2-chamber view, namely the anterior atrial wall, the side of the bicuspid ring free wall, and the top of atrium; three reference points were placed on the 4-chamber view, namely the side of the bicuspid ring septum, the side of the bicuspid ring free wall, and the top of atrium; three cardiac cycles were manually tracked to obtain a stable strain rate curve, and the LA sampling observation point was placed on the image of LAVmax to obtain the total LA strain (Ƹ) and LA strain rate curve. This measurement method was confirmed in our previous study [12,14]. The curve consists of an upward waveform – ventricular systolic strain rate (SRs) and two downward waveforms – early ventricular diastolic strain rate (SRe) and atrial systolic strain rate (SRa), where Ƹ and SRs reflect the atrial reserve function, SRe reflects the atrial ventricular diastole function, and SRa reflects the atria pump function.

### 2.6 Arrhythmia recurrence assessment

All patients were treated with antiarrhythmic drugs and anticoagulants for 3 months after surgery. Regular dosing of blood pressure medications was maintained, and no additional medications were prescribed. If no thrombus ≥5 mm related to the device or residual shunt around the device was found in the TEE examination at 3 months, patients receiving the combined treatment were advised to receive the dual antiplatelet treatment (aspirin [100 mg/day] ± clopidogrel [75 mg/day]) for another 3 months while orally taking aspirin until 1 year after surgery. Patients underwent 24 h dynamic electrocardiogram monitoring at 3 and 12 months after surgery to assess the presence of atrial arrhythmia. AF, atrial flutter, or atrial tachycardia that occurred 3 months after ablation and lasted ≥30 s were considered recurrence of AF.

### 2.7 Statistical analysis

Statistical analysis was conducted using SPSS Statistics, version 25 (IBM, Armonk, NY, USA). The Kolmogorov–Smirnov test was applied to determine whether the data followed a normal distribution. Normally distributed data are expressed as the mean ± standard deviation, while non-normally distributed data are presented as medians with interquartile ranges. Counting data are expressed as percentages (%). Student’s *t*-test (normality) or Mann–Whitney *U* test (non-normality) and chi-squared test were used to compare the baseline parameters between the two groups of patients. Continuous data of different indices after the operation were assessed with analysis of variance for repeated measures. To compare LA function between the two groups, we used the least significant difference test. A *P*-value of <0.05 was considered statistically significant. PSM analysis (1:1 matching) using age, sex, CHA2DS2-VASc score, and HAS-BLED score was performed to control for selection bias within a caliper of 0.05 on the propensity-score using SPSS 23 software.

## 3 Results

### 3.1 Baseline data

Among the 178 patients in this study, 89 patients underwent PVI combined with Watchman LAAC (combined treatment group) successfully, and 89 patients in the control group underwent CA only (simple CA group). Clinical features are shown in Table 1. There were no statistical differences in the age, gender, BMI, CHA 2 DS 2-VASc, HAS-BLED score, complications, drugs, and LAA type before ablation between the combined treatment group and the matched AF ablation group (*P* > 0.05). Results are shown in Table 1.

**Table 1:** Baseline characteristics of the population

	Treatment group	Control group	*P*-value
Age (years)	64.17 ± 7.56	65.60 ± 8.15	0.23
Sex, male, *n* (%)	52 (58)	44 (49)	0.23
Smoke, *n* (%)	19 (21)	15 (17)	0.45
Alcohol, *n* (%)	10 (11)	12 (14)	0.65
Hypertension, *n* (%)	62 (70)	68 (76)	0.31
Coronary artery disease, *n* (%)	44 (49)	45 (51)	0.88
Diabetes mellitus, *n* (%)	24 (27)	28 (32)	0.51
Congestive heart failure, *n* (%)	41 (46)	32 (36)	0.17
II	31 (35)	28 (32)	0.63
III	9 (10)	4 (5)	0.15
IV	1 (1)	0 (1)	0.32
Stroke, *n* (%)	50 (56)	46 (52)	0.55
Bleeding, *n* (%)	2 (2)	2 (2)	1
BMI	25.76 ± 2.97	26.05 ± 4.06	0.59
serum creatinine	72.26 ± 20.28	72.84 ± 22.99	0.87
CHA2DS2-VASc score	4.16 ± 1.56	4.20 ± 1.69	0.84
HAS-BLED score	2.96 ± 0.92	3.10 ± 0.72	0.30
**Drugs before ablation**, *n* (%)			
AAD	12 (13)	10 (11)	0.65
Beta-blocker	32 (36)	36 (40)	0.54
ACEI or ARB	27 (30)	25 (28)	0.74
Aldosterone receptor antagonists	6 (7)	5 (6)	0.76
Other diuretics	8 (9)	6 (7)	0.58
**LAA type**, *n* (%)			
Chicken wing type	40 (45)	38 (43)	0.76
Cauliflower type	32 (36)	35 (39)	0.64
Arrowhead type	17 (19)	16 (18)	0.85

Data are shown as mean ± SD or *n* (%). BMI, body mass index; AAD, antiarrhythmic drugs; ACEI, angiotensin converting enzyme inhibitor; ARB, angiotensin-2 receptor blockade.

### 3.2 Intra-group comparison of left atrial functions between the two groups

Among a total of 178 patients, 37 patients were excluded due to AF or atrial flutter on echocardiography at follow-up, the remaining 141 patients (69 in the combined treatment group and 72 in the simple CA group) were included in the analysis of LA structure, Ƹ, and SR.

**Figure 3:**
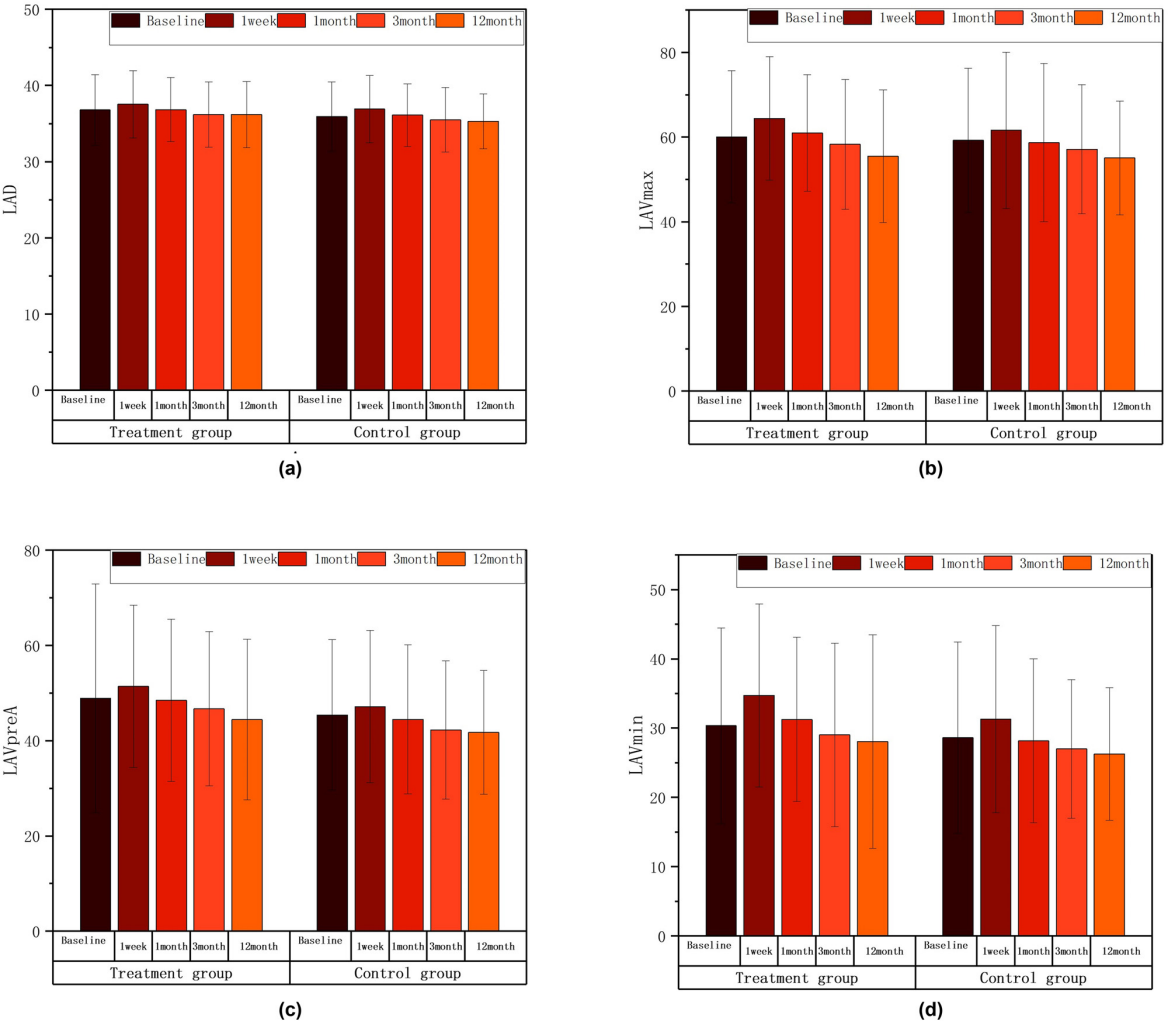
LAD, LAVmax, LAVpreA, LAVmin intra-group comparison diagram; ∗*P* < 0.05; ∗∗*P* < 0.01; ∗∗∗*P* < 0.001 compared with the baseline level. There were no statistical differences in these parameters in all groups when compared with those before surgery. The postoperative comparison of parameters representing left atrial structure, such as LAD (a), LAVmax (b), LAVpreA (c), and LAVmin (d), did not show any statistically significant differences when compared to the preoperative values in both groups.

LAD, LAVmax, LAVpreA, and LAVmin values in the combined treatment group and simple CA group increased at 1 week compared with that before surgery, and gradually decreased from 1 month, and these parameters were similar to or slightly below the baseline levels at 12 months after ablation. There were no statistical differences in these parameters in all groups when compared with those before surgery. Results are shown in Table 2 and Figure 3.

**Table 2:** Evolution of LA function after the LAAC combined with CA and simple CA group assessed volumetric indices

Variables	Groups	Baseline	1 W	1 M	3 M	12 M	*P*-value
LA D	Treatment group	36.80 ± 4.61	37.54 ± 4.41	36.83 ± 4.20	36.18 ± 4.29	36.20 ± 4.33	0.003
	*P* for time		0.341	0.975	0.412	0.432	
	Control group	35.93 ± 4.55	36.90 ± 4.41	36.11 ± 4.11	35.49 ± 4.23	35.29 ± 3.59	0.001
	*P* for time		0.196	0.802	0.547	0.354	
LAVmax	Treatment group	60.05 ± 15.63	64.39 ± 14.58	60.95 ± 13.78	58.30 ± 15.32	55.48 ± 15.69	<0.001
	*P* for time		0.101	0.724	0.519	0.097	
	Control group	59.22 ± 17.07	61.61 ± 18.47	58.69 ± 18.69	57.15 ± 15.26	55.07 ± 13.45	0.011
	*P* for time		0.422	0.860	0.444	0.107	
LAVpreA	Treatment group	48.91 ± 24.03	51.42 ± 17.02	48.49 ± 17.02	46.70 ± 16.20	44.44 ± 16.88	0.021
	*P* for time		0.486	0.909	0.535	0.216	
	Control group	45.41 ± 15.78	47.18 ± 15.95	44.48 ± 15.64	42.26 ± 14.53	41.73 ± 12.98	0.104
	*P* for time		0.519	0.730	0.224	0.135	
LAVmin	Treatment group	30.36 ± 14.10	34.73 ± 13.23	31.26 ± 11.85	29.02 ± 13.26	28.08 ± 15.41	0.001
	*P* for time		0.069	0.694	0.572	0.375	
	Control group	28.64 ± 13.81	31.32 ± 13.50	28.19 ± 11.81	27.00 ± 10.01	26.27 ± 9.56	0.008
	*P* for time		0.241	0.836	0.416	0.235	

Data are shown as mean ± S. *P* for time is the *P* value of the relevant parameters compared with their baseline values at a single follow-up time point.

**Figure 4:**
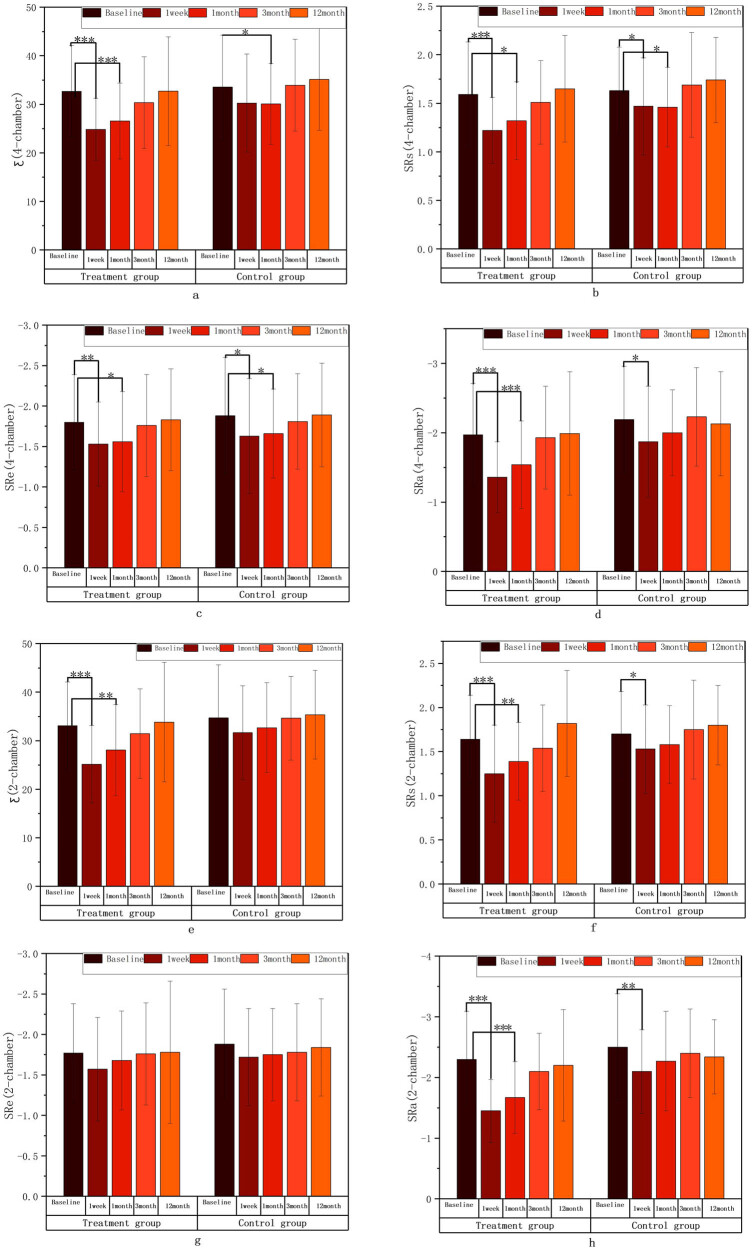
Reserve (SRs [2-chamber (f), 4-chamber (b)]), ventricular diastole (SRe [2-chamber (g), 4-chamber (c)]), and pump (Ƹ [2-chamber (e), 4-chamber (a)], SRa [2-chamber (h), 4-chamber (d)]) functions of the LA intra-group comparison diagram; ∗*P* < 0.05; ∗∗*P* < 0.01; ∗∗∗*P* < 0.001 compared with the baseline level.

The values of Ƹ, SRs, SRe, and SRa in the 2-chamber and 4-chamber views in the two groups decreased at 1 week compared with that before surgery, and gradually increased from 1–3 months to 12 months with a consistent trend in the two groups, and these parameters were similar to or slightly below the baseline levels at 12 months after ablation. Compared with those before surgery, Ƹ (4-chamber), SRs (4-chamber), the absolute value of SRa (4-chamber), Ƹ (2-chamber), SRs (2-chamber), and the absolute value of SRa (2-chamber) in the combined treatment group significantly decreased at 1 week (*P* < 0.001), the absolute value of SRe (4-chamber) in the combined treatment group and the absolute value of SRa (2-chamber) in the simple CA group significantly decreased at 1 week (*P* < 0.01), and SRs (4-chamber), the absolute values of SRe (4-chamber), the absolute values of SRa (4-chamber), and SRs (2-chamber) in the simple CA group decreased at 1 week (*P* < 0.05). Compared with those before surgery, Ƹ (4-chamber), the absolute value of SRa (4-chamber), and the absolute value of SRa (2-chamber) in the combined treatment group significantly decreased at 1 month (*P* < 0.001); Ƹ (2-chamber) in the combined treatment group and SRs (2-chamber) in the simple CA group significantly decreased at 1 month (*P* < 0.01); SRs (4-chamber) and the absolute value of SRe (4-chamber) in the combined treatment group and Ƹ (4-chamber), SRs (4-chamber), and the absolute value of SRe (4-chamber) in the simple CA group significantly decreased at 1 month (*P* < 0.05). There were no statistical differences in the Ƹ, SRs, SRe, and SRa values on 2-chamber and 4-chamber views between the two groups at 3 and 12 months when compared with those before surgery. Results are shown in Table 3 and Figure 4.

**Table 3:** Evolution of LA function after the LAAC combined with CA and simple CA group assessed strain indices

Variables	Groups	Baseline	1 W	1 M	3 M	12 M	*P*-value
Ƹ (4-chamber)	Treatment group	32.67 ± 9.41	24.85 ± 6.35	26.57 ± 7.81	30.35 ± 9.45	32.71 ± 11.20	＜0.001
	*P* for time		＜0.001	＜0.001	0.151	0.980	
	Control group	33.56 ± 10.72	30.25 ± 10.09	30.07 ± 8.33	33.93 ± 9.46	35.13 ± 10.47	＜0.001
	*P* for time		0.059	0.031	0.824	0.376	
SRs (4-chamber)	Treatment group	1.59 ± 0.54	1.22 ± 0.34	1.32 ± 0.40	1.51 ± 0.43	1.65 ± 0.55	＜0.001
	*P* for time		＜0.001	0.01	0.336	0.519	
	Control group	1.63 ± 0.45	1.47 ± 0.50	1.46 ± 0.41	1.69 ± 0.54	1.74 ± 0.44	＜0.001
	*P* for time		0.040	0.020	0.482	0.151	
SRe (4-chamber)	Treatment group	−1.80 ± 0.59	−1.53 ± 0.52	−1.56 ± 0.62	−1.76 ± 0.63	−1.83 ± 0.63	＜0.001
	*P* for time		0.037	0.047	0.538	0.922	
	Control group	−1.88 ± 0.72	−1.63 ± 0.71	−1.66 ± 0.55	−1.81 ± 0.59	−1.89 ± 0.64	0.001
	*P* for time		0.003	0.002	0.32	0.90	
SRa (4-chamber)	Treatment group	−1.97 ± 0.74	−1.36 ± 0.51	−1.54 ± 0.63	−1.93 ± 0.74	−1.99 ± 0.89	＜0.001
	*P* for time		＜0.001	＜0.001	0.738	0.895	
	Control group	−2.19 ± 0.77	−1.87 ± 0.80	−2.00 ± 0.62	−2.23 ± 0.71	−2.13 ± 0.75	0.001
	*P* for time		0.016	0.106	0.729	0.641	
Ƹ (2-chamber)	Treatment group	33.09 ± 9.01	25.14 ± 7.95	28.10 ± 9.32	31.45 ± 9.23	33.83 ± 12.30	＜0.001
	*P* for time		＜0.001	0.002	0.293	0.688	
	Control group	34.72 ± 10.89	31.65 ± 9.68	32.68 ± 9.25	34.64 ± 8.65	35.35 ± 9.15	0.012
	*P* for time		0.076	0.227	0.960	0.710	
SRs (2-chamber)	Treatment group	1.64 ± 0.50	1.25 ± 0.55	1.39 ± 0.44	1.54 ± 0.49	1.82 ± 0.60	＜0.001
	*P* for time		＜0.001	0.02	0.203	0.072	
	Control group	1.70 ± 0.48	1.53 ± 0.50	1.58 ± 0.44	1.75 ± 0.56	1.80 ± 0.45	＜0.001
	*P* for time		0.046	0.136	0.538	0.179	
SRe (2-chamber)	Treatment group	−1.77 ± 0.61	−1.57 ± 0.64	−1.68 ± 0.61	−1.76 ± 0.63	−1.78 ± 0.88	0.004
	*P* for time		0.064	0. 357	0.931	0.962	
	Control group	−1.88 ± 0.68	−1.72 ± 0.60	−1.75 ± 0.57	−1.78 ± 0.60	−1.84 ± 0.60	0.213
	*P* for time		0.143	0.235	0.363	0.706	
SRa (2-chamber)	Treatment group	−2.30 ± 0.79	−1.45 ± 0.52	−1.67 ± 0.59	−2.10 ± 0.63	−2.20 ± 0.92	＜0.001
	*P* for time		＜0.001	＜0.001	0.104	0.505	
	Control group	−2.50 ± 0.88	−2.10 ± 0.69	−2.27 ± 0.82	−2.40 ± 0.73	−2.34 ± 0.61	＜0.001
	*P* for time		0.003	0.111	0.455	0.227	

Data are shown as mean ± S. *P* for time is the *P* value of the relevant parameters compared with their baseline values at a single follow-up time point.

### 3.3 Comparison of left atrial functions between two groups

**Figure 5:**
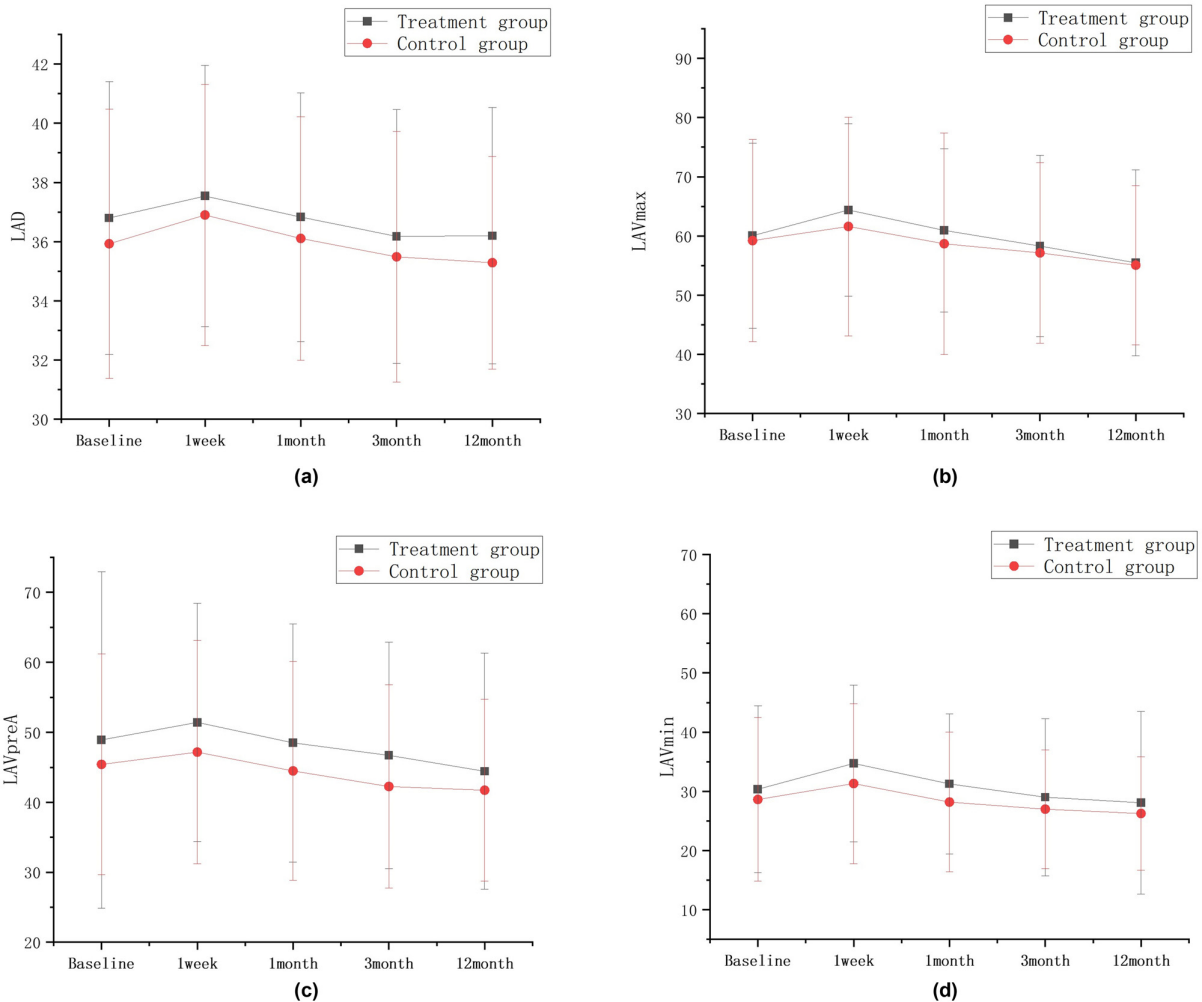
Left atrial structure LAD (a), LAVmax (b), LAVpreA (c), and LAVmin (d) trend chart.

**Figure 6:**
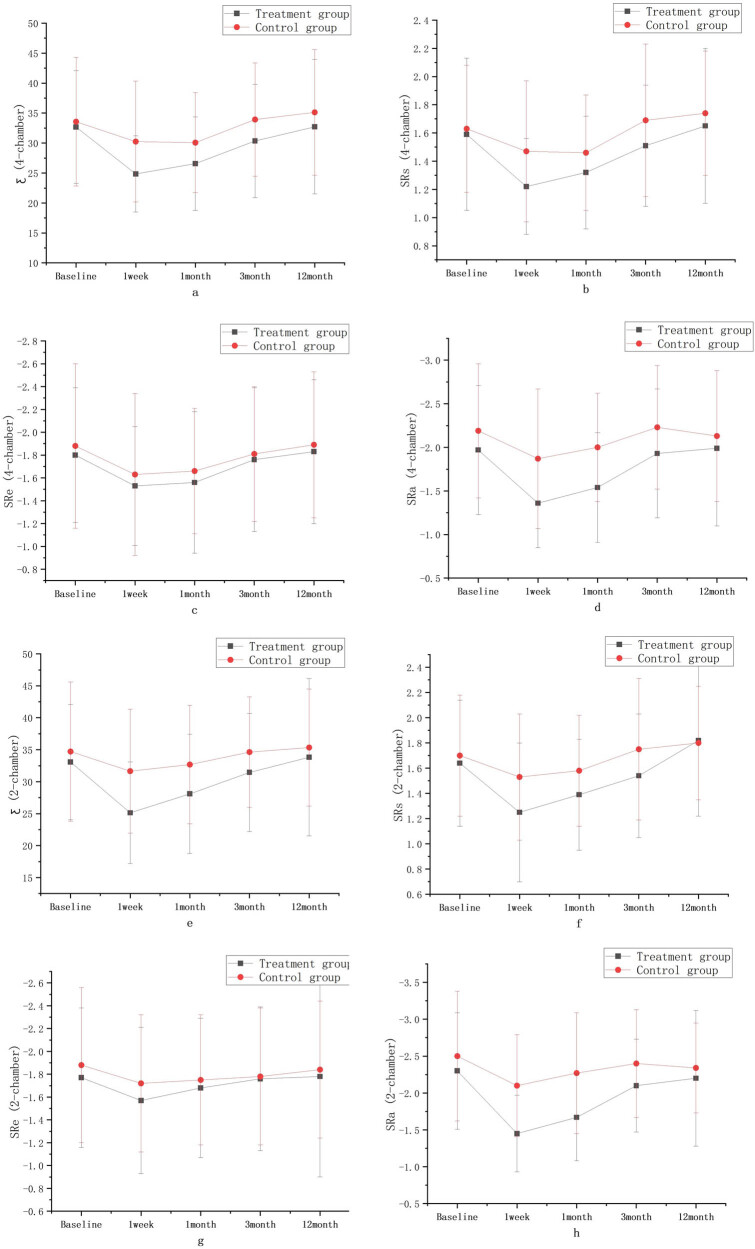
Reserve (SRs [2-chamber (f), 4-chamber (b)]), ventricular diastole (SRe [2-chamber (g), 4-chamber (c)]), and pump (Ƹ [2-chamber (e), 4-chamber (a)], SRa 2-chamber (h), 4-chamber (d)]) functions of the LA trend chart.

There was consistency in the changes of the structural function (LAD, LAVmax, LAVpreA, LAVmin), the reserve function (Ƹ, SRs), the ventricular diastole function (SRe), and the pump function (SRa) of LA in patients in the combined treatment group and simple CA group over time. Results are shown in Figures 5 and 6.

**Figure 7:**
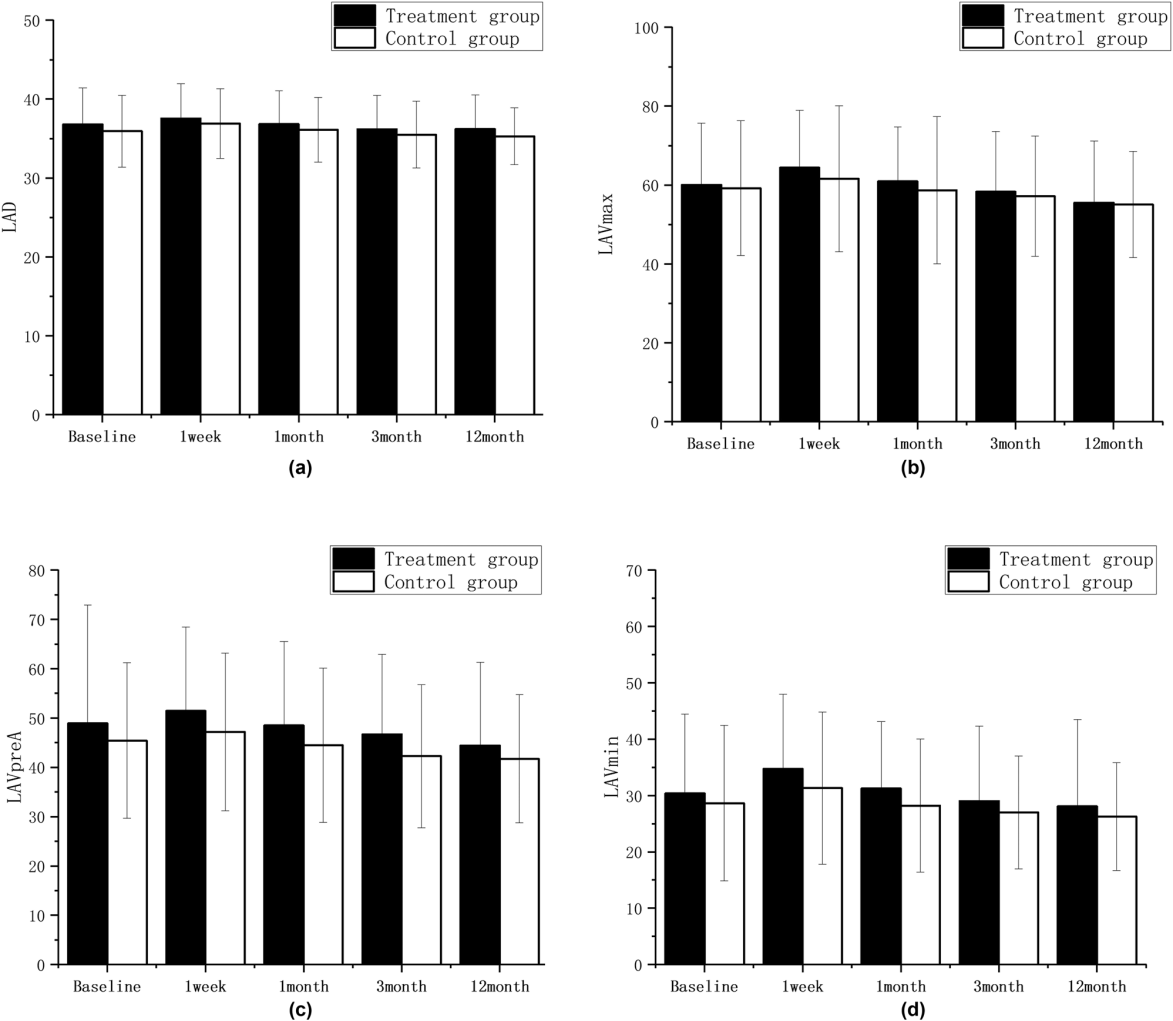
LAD (a), LAVmax (b), LAVpreA (c), and LAVmin (d) comparison diagram between groups; ∗*P* < 0.05; ∗∗*P* < 0.01; ∗∗∗*P* < 0.001 vs control group.

**Figure 8:**
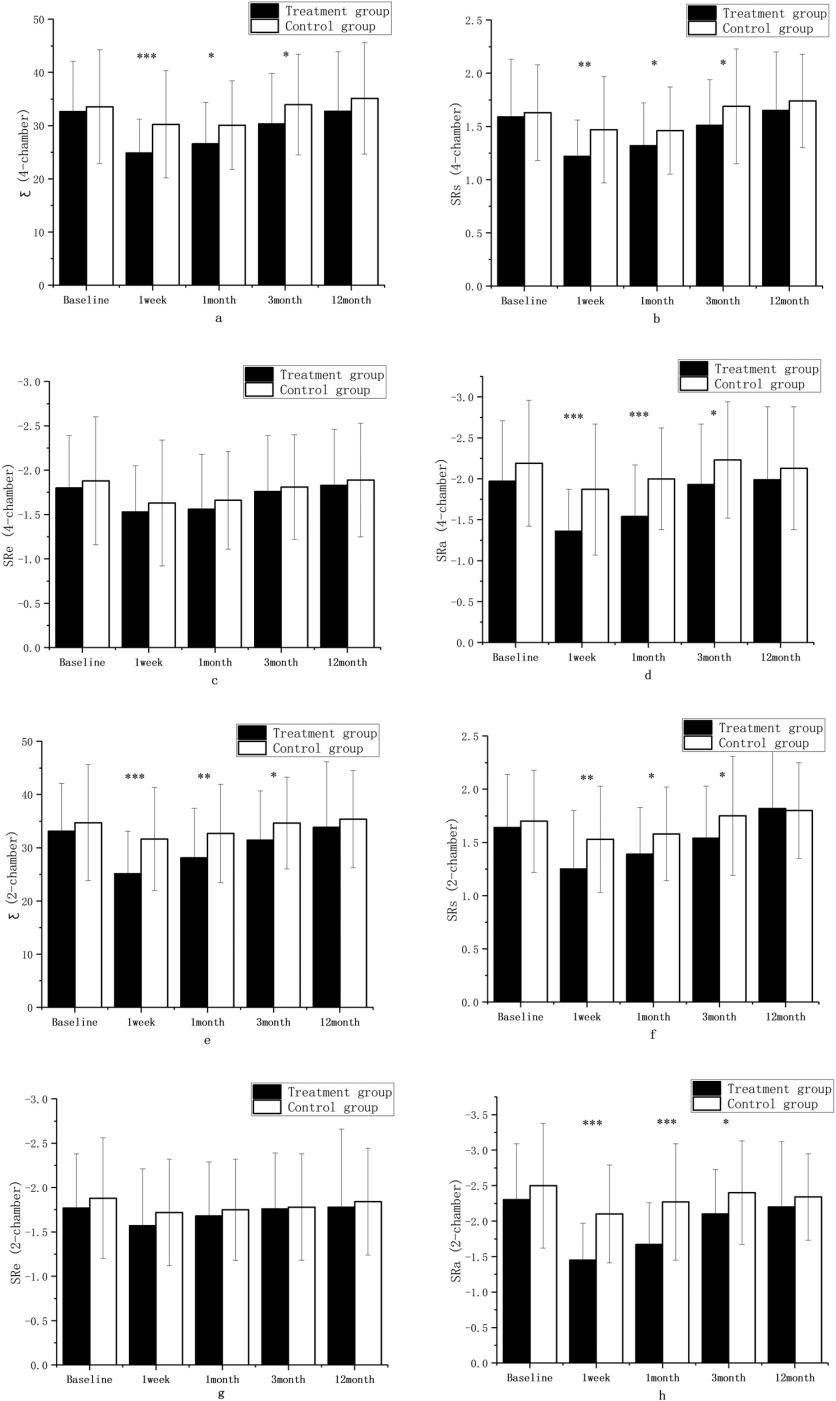
Ƹ (4-chamber) (a), SRs(4-chamber) (b), SRe (4-chamber) (c), SRa (4-chamber) (d), Ƹ (2-chamber) (e), SRs (2-chamber) (f), SRe (2-chamber) (g), SRa (2-chamber) (h) comparison diagram between groups; ∗*P* < 0.05; ∗∗*P* < 0.01; ∗∗∗*P* < 0.001 vs control group.

The structural functions of LA structure between the two groups (LAD, LAVmax, LAVpreA, LAVmin) and channel function (SRe (4-chamber), SRe (2-chamber) were not statistically significant between the two groups at each time point. The atrial catheter function (ventricular diastole function) (Ƹ [4-chamber], Ƹ [2-chamber], SRs [4-chamber], SRs [2-chamber]) and atrial pump function (SRa [4-chamber], SRa [2-chamber]) during the follow-up at 1 week, 1 month, and 3 months were significantly different between the two groups, and the values were lower in the combined treatment group than in the simple CA group. There were no statistical differences in the reserve function (Ƹ [4-chamber], SRs [4-chamber], Ƹ [2-chamber], SRs [2-chamber]), and the pump function (SRa [4-chamber], SRa [2-chamber]) between the two groups at the 12-month follow-up. Results are shown in Figures 7 and 8.

### 3.4 Clinical outcome

All surgeries were successful, and all implants were well sealed (residual shunt <5 mm) in the combined treatment group. Three patients in the combined treatment group and one patient in the simple CA group developed pericardial effusion during the surgery; however, they recovered normally after percutaneous drainage. During the 3-month follow-up, one patient in the combined treatment group was found to have erythrocyte spontaneous contrast in the LA using TEE, and one patient with recurrent AF in the simple CA group developed ischemic stroke at 6 months. In the combined treatment group, one patient developed severe cerebral hemorrhage at 3 months, one patient had gingival bleeding at 9 months, and one patient had pseudoaneurysm at 1 month, and no bleeding death was reported. In the simple CA group, one patient developed a small amount of hemoptysis during surgery, one patient had hematoma of the groin 10 days after surgery, and one patient with cerebral hemorrhage 2 months after AF ablation recovered after conservative treatment. All patients underwent pulmonary vein isolation to restore sinus rhythm. During the 12-month follow-up, no device dislocation occurred; 20 patients (23%) in the combined treatment group and 17 patients (19%) in the simple CA group developed recurrent AF or atrial flutter. There was no statistical difference between the two groups.

## 4 Discussion

In a retrospective, observational matched pair study [14], LAAC did not impair the left atrial systolic function or the left atrial ejection fraction, compared with patients with untreated LAA, while the echocardiography at follow-up showed that the reserve and ventricular diastole functions of LA were significantly impaired in both groups. Dar et al. [15] and Murtaza et al. [16] studied patients implanted with Lariat and Watchman occluders using Doppler imaging and STE and found no improvement in the reserve and ventricular diastole functions of LA after LAAC. The results of some studies suggest that LAAC causes no significant changes in the LA and left heart functions although LAA accounts for 10% or so of the total volume of LA [17]. Other studies also support the contention that there is no negative effect on the heart function after LAA excision [18]. The study includes both endocardial and epicardial LAAC, which can impact the assessment of left atrial function. However, the effect of LAAC on the left atrial function in patients with different types of AF have not been reported as yet.

In our study, among the two groups of patients, the most common LAA type was the chicken wing type, followed by the cauliflower type, with the arrowhead type being less common. This distribution aligns with previous research findings [19]. Known for the pathological connection to AF, the LAA is the most common source of thromboembolism in patients with AF and may be an arrhythmogenic source for the maintenance of AF. Potential interventions of the LAA for stroke prevention have recently been developed through better understanding its anatomy and physiology. LAAC is an alternative to the use of life-long anticoagulation in selected NVAF cases. Among participants with AF who had undergone cardiac surgery, most of whom continued to receive ongoing antithrombotic therapy, the risk of ischemic stroke or systemic embolism was lower with concomitant LAAC performed during the surgery than without it [20]. However, the LAA is structurally complex and has considerable morphological variations among individuals, and it can be challenging to generalize the device for all patients. Continued technological developments including occlusion/ligation through epicardial, endocardial, or surgical approaches, as well as operator expertise regarding LAA anatomy, physiology, and pathophysiology, should improve interventional outcomes. The absence of trials comparing each LAAC modality, as with the novel anticoagulant therapy, makes it difficult to decide which therapy is superior for patients. Since each LAAC technique offers unique benefits and risks, patient selection must be individualized. Anatomical recognition of the location of the LAA ostium, definition and management of device leaks, and management of LAA with variant anatomy remain important topics to be addressed [21]. The neck of the LAA is a smooth-walled, truncated cone-shaped channel located between its pectinated component and the body of the LA. Notably, the current research found that there are no statistically significant differences or correlations regarding the LAA type and the LAA neck morphometry. Precise definition and morphometrical details of the LAA neck that were introduced in previous study may influence the effectiveness and safety of LAA exclusion procedures [22]. LAAC can be performed via epicardial access or endocardial access with an occlusion device. Endocardial and epicardial LAAC techniques shows comparable implantation outcomes and safety profile and stroke prevention in patients with AF [23]. The number of LAAC is systematically increasing worldwide. Unfortunately, LAAC, like any invasive procedure, carries a risk of complications. One of the worst, but rare, complications of LAAC is coronary artery damage. The study showed that most dangerous distances (30.2%) occurred in the LAA landing zone dimension. The data showed that landing zones more distal from the orifice of the LAA are safer in terms of the circumflex artery damage. Therefore, LAAC should always be performed with caution, to avoid iatrogenic complications [24].

In this study, changes in the reserve, ventricular diastole, and pump functions of LA over time were consistent in the two groups – the functions decreased at 1 week and gradually recovered at 1 month, and there were no significant differences when compared with the values before surgery at the 12-month follow-up. The reasons may be that the heat effect of CA may cause damage and necrosis to cardiac muscle tissue in LA, which leads to edema and scar tissue formation, followed by secondary conduction dysfunction that leads to non-uniform contraction of atrial muscle tissue, transient increase of LAD, and transient decrease of LA ejection fraction; finally, transient decline in LA functions and atrial stunning occur. However, these changes in LA functions are reversible and recoverable. An aberrant state of LA electro-mechanical connection was produced by atrial stunning 1 week after surgery, and it was gradually restored within 3 months [25]. Our previous studies indicated that patients with paroxysmal AF in the three groups showed significant decrease in the total LA strain (εP), LA reserve function strain (SRP), LA ventricular diastole function strain (SRE), and LA pump function strain (SRA) on the 2-chamber and 4-chamber views at 7 days after ablation [26], which suggested left atrial stunning; these parameters gradually recovered within 4 weeks to 3 months after ablation, and were completely restored to the baseline levels within 9 months after ablation. Our findings of the changes in the left atrial functions are in line with the results of previous studies [26,27,28]. Given that thromboembolic events are linked to atrial stasis, we emphasized the significance of appropriate anticoagulant therapy in both groups 3 months after surgery.

The results of our previous studies suggested that patients with persistent AF in the combined treatment group and the simple CA group started to show significant improvement in the left atrial functions at 1 week, which continued till 3 months and maintained till 12 months [10]. In this study, the difference was that the left atrial functions of patients with paroxysmal AF in the two groups significantly decreased at 1 week and 1 month after the surgery when compared with the baseline levels, and were gradually restored to the preoperative levels at 3 months and remained at 12 months. The discrepancy between studies may be due, in large part, to the fact that various forms of AF have varying impacts on hemodynamics following one-stop surgery [10]. Prior to surgery, the left atrial function of most patients with paroxysmal AF was significantly higher than that of patients with persistent AF. Patients with paroxysmal AF tended to have more normal left atrial function at baseline, suggesting that there was less room for improvement in left atrial function following ablation. Patients who have persistent AF, on the other hand, experience the opposite. Left atrial enlargement is evident in patients with persistent AF prior to surgery, and left atrial function is typically below normal at baseline; however, left atrial function improves more significantly after ablation. The different responses of left atrial functions to ablation can be explained as follows. AF ablation on atrial myocardium has an inherently detrimental effect on LA functions, and the atrial function impairment may last 2–3 months while leading to LA reverse remodeling by reducing the AF burden [29]. This beneficial effect is most obvious in patients with persistent AF with high AF burden and impaired baseline functions [30]. Thus, the effect of AF ablation on LA function depends on the balance of these detrimental and beneficial effects. Several studies have revealed that patients with persistent AF who recovered their sinus rhythm after successful CA, developed significant LA reverse remodeling in the long term [31–33]. A further comparative study showed that patients with persistent AF had significant improvement in the left atrial functions than those with paroxysmal AF after ablation [28].

On the other hand, additional LAAC influenced the reserve and ventricular diastole functions of LA within 3 months among patients with persistent AF who underwent one-stop surgery, while the results of our study showed that the reserve and pump (contraction) functions of LA were affected by LAAC within the first 3 months. The reasons for this may be that with faster LAA emptying and better LA and LAA contraction functions in patients with paroxysmal AF than in those with persistent AF, the paroxysmal AF contraction functions may be affected more severely in early LAAC. Previous studies have indicated that patients with paroxysmal AF have better LA and LAA contraction functions, and LAAC leads to a dramatic decrease in LA compliance in the short term, followed by the increase in the size and pressure of LA and decrease in the cardiac output, resulting in decrease in the LA reserve function, which may influence the reserve and contraction functions of LA in the short and medium term.

Our study’s strength lies in its ability to elucidate the effect of LAAC on the left atrial functions after eliminating the effect of CA by comparing patients in the one-stop surgery group with those in the simple CA group. During the 3-month follow-up, and especially at 1 week and 1 month, patients in the combined treatment group had significantly decreased reserve function (Ƹ, SRs) and pump function (SRa) of LA compared with the simple CA group, and the difference was statistically significant at 3 months; there was no difference in the left atrial functions after 3 months. The causes may be as follows. First, LAA has some important functions with its unique anatomical and physiological properties, and these functions and their contribution to cardiac hemodynamics have been confirmed. Besides, due to the higher compliance than LA and an important role in the presence of LA pressure and/or volume overload [34], LAA may affect the LA reserve and contraction functions in the short term after LAAC. The mechanical properties of the LAA are determined by its contractile characteristics, playing a role in hemodynamic function by regulating left atrial pressure. As a part of the LA, the LAA possesses a certain capacity and is structurally more pliable than the rest of the LA. When there is an overload in volume or pressure within the LA, the LAA can expand to become an important reservoir, alleviating pressure in the LA and ensuring adequate blood filling of the left ventricle [35]. Second, LAA has little contribution to the stroke volume, thus LAAC is of little significance in the Frank–Starlin mechanism. The Frank–Starlin mechanism describes the relationship between the increased length of myocardial fiber and its mechanical property, showing an essential therapeutic and prognostic value in the evaluation of left atrial functions. Previous studies indicated that the Frank–Starling mechanism was also found in the LA [36]. The LA is the reservoir, ventricular diastole, and booster pump that returns blood from the lungs to the heart. In previous studies, as the LA preload increased, the LA contractility increased to a certain extent and then decreased, which indicated that there was a Frank–Starlin mechanism. Using 2D/3D TEE (TTE and 2D STI), Coisne et al. [37] found that the reserve and contraction functions of LA, such as LA volume index, LA reserve volume, LAEF, and LA strain, were improved after one-stop surgery. This may be attributed to the Frank–Staring compensatory mechanism rather than the changes in the intrinsic myocardial properties. Our study used patients with AF treated solely with radiofrequency ablation as the control group. We found that the additional LAAC on top of CA had a short-term impact on the left atrial reservoir and pump function in patients with paroxysmal AF, which gradually returned to normal.

## 5 Limitations

There are limitations to this study. Due to the low number of patients treated with LAAC at our center, a single control group was unavailable to directly indicate the efficacy of LAAC on LA functions. We observed no significant changes in the volume and strain parameters at 12 months when compared with that before surgery, thus only long-term data can reveal whether the result is persistent or transient. Therefore, these deficiencies should be considered and further investigated. Study was not randomized, inclusion criteria differed in important parts and lack of some data which was declared to be measured may lead to data bias. Additionally, it is suspected that the population was ethnically homogenous. Additionally, only one type of LAAC procedure was used.

## 6 Conclusion

The left atrial functions decreased in the two groups at 1 week and 1 month, but they were close to or restored to the preoperative levels at 3 months. When compared with the simple CA group, patients in the combined treatment group showed a significant decrease in the reserve function and pump functions of the LA within the first 3 months; however, there were no statistical differences at 12 months between the two groups, suggesting that LAAC may have partial short-term impact on LA reserve function and pump function of patients with paroxysmal AF; these functions were later restored gradually, and were not affected at 12 months.
